# Elevated hyaluronic acid levels in severe SARS-CoV-2 infection in the post-COVID-19 era

**DOI:** 10.3389/fcimb.2024.1338508

**Published:** 2024-02-08

**Authors:** Yanyan Li, Xinyu Cui, Na Zhu, Yingying Lin, Xin Li

**Affiliations:** ^1^ Center of Integrative Medicine, Beijing Ditan Hospital, Capital Medical University, Beijing, China; ^2^ Center of Integrative Medicine, Peking University Ditan Teaching Hospital, Beijing, China

**Keywords:** hyaluronic acid, severe acute respiratory syndrome coronavirus-2, coronavirus disease 2019, severe infection, lung involvement, progression

## Abstract

**Objective:**

Human identical sequences of severe acute respiratory syndrome coronavirus-2 (SARS-CoV-2) promoted the coronavirus disease 2019 (COVID-19) progression by upregulating hyaluronic acid (HA) via NamiRNA-enhancer network, based on previous experimental research. This study aimed to investigate the predictive value of HA for the severity of SARS-CoV-2 infection in the post-COVID-19 era.

**Methods:**

A total of 217 consecutive patients with COVID-19 were enrolled at Beijing Ditan Hospital between July 2023 and October 2023. HA levels were analyzed using biochemical detector. Logistic regression analysis was used to screen independent factors for severe COVID-19. The predictive performance of HA for severe infection was assessed by ROC curve. Furthermore, the relationship between HA levels and COVID-19 severity was investigated using multivariate logistic regression models after adjustment for potential confounders.

**Results:**

According to the cut-off value of HA, COVID-19 patients were divided into HA < 90 ng/mL group (80 cases) and HA ≥ 90 ng/mL group (137 cases). High HA levels were positively associated with the severe SARS-CoV-2 infection, including elevated inflammatory indicators, severe lung involvement, prolonged clinical course, and higher incidence of respiratory failure and death (P < 0.05). Logistic regression analysis suggested that HA was an independent predictor of severe COVID-19 (OR = 4.540, 95% CI = 2.105-9.790, P < 0.001). ROC curve analysis showed that the AUC of HA for severe infection was 0.724. HA levels were significantly higher in COVID-19 cases compared to the healthy population (123.9 (82.6, 174.1) vs. 50.5 (37.8, 66.8), P < 0.001), but similar to those with non-SARS-CoV-2 lung infection (121.6 (78.5, 175.6) vs. 106.0 (66.5, 149.7), P = 0.244). We also found that the first COVID-19 infections had higher HA levels (118.8 (79.5, 174.3) vs. 85.0 (61.1, 128.8), P < 0.001) and a higher proportion of severe infection (37.1% vs. 21.3%, P = 0.043) than re-infections. However, HA expression failed to fully return to normal levels with infection recovery (204.7 (152.9, 242.2) vs. 97.0 (69.3, 137.3), P < 0.001).

**Conclusion:**

HA was associated with severe SARS-CoV-2 infection and could be used as a novel serum biomarker to predict the risk of COVID-19 progression in the post-COVID-19 era.

## Introduction

Coronavirus disease 2019 (COVID-19), caused by severe acute respiratory syndrome coronavirus-2 (SARS-CoV-2), has placed a significant burden on public health over the past 3 years (Berlin et al., 2020). The harmful effects of this insidious virus have been significantly reduced by the development and application of vaccines and therapeutics, but its rapid mutation and high transmission still pose a threat to humans (Gottlieb et al., 2022). A significant proportion of the population, especially the elderly in poor health, remain at high risk of severe COVID-19 (Bash et al., 2023). A prominent sign of severe SARS-CoV-2 infection is the progression of lung lesions, such as ground-glass opacities, pulmonary consolidation and acute respiratory distress syndrome (ARDS) (Xu et al., 2020). Although chest CT can early identify patients at risk of COVID-19 progression, its use may be relatively limited by poor compliance and cost. Of note, most of people prefer serological assessment of infection severity to chest CT scans, especially in the outpatient setting. Thus, finding a simple and feasible serum biomarker to complement chest CT to assist clinicians in the early identification of high-risk individuals is necessary and cost-effective in the post-COVID-19 era.

Hyaluronic acid (HA) is an important component of the extracellular matrix involved in various biological and pathological processes, including tissue injury and repair, immune responses and inflammatory reactions (Liang et al., 2016; Zheng et al., 2023). Accumulating evidence suggests that HA plays an important role in the inflammatory process, it could be considered an inflammatory biomarker. The presence of HA in inflamed airways has been related to several infectious diseases, including influenza and sepsis (Lauer et al., 2015; Bell et al., 2019). Recent experimental investigation has identified five identical sequences between SARS-CoV-2 and the human genome that can activate hyaluronan synthase 2 via the NamiRNA-enhancer network, thereby increasing HA expression (Li et al., 2022). ARDS is one of the clinical manifestations of critically ill COVID-19 cases, and has been shown to be associated with an accumulation of HA in the airways (Albtoush and Petrey, 2022). Autopsy evidence indicated that HA was present in the alveolar spaces of the lungs of deceased COVID-19 cases (Hellman et al., 2020). Subsequent clinical studies have suggested that HA is associated with the severity of COVID-19, and may serve as a potential therapeutic target (Yang et al., 2022).

In the post-COVID-19 era, the epidemiological characteristics of COVID-19 have undergone a number of changes, including increased population immunity, reduced viral virulence and increased risk of repeat infections. Whether there is a positive association between HA and COVID-19 progression in clinical practice is unclear. This study aimed to investigate the predictive ability of HA for severe SARS-CoV-2 infection, and to provide clinical evidence to support studies targeting HA in inflammatory respiratory disease.

## Materials and methods

### Patient population

This is a prospective clinical study. From July 2023 to October 2023, 217 patients with COVID-19 were consecutively enrolled at the Infection Centre of Beijing Ditan Hospital. Exclusion criteria included (1) severe non-infectious pulmonary disease, such as interstitial pneumonia and pulmonary edema; (2) patients with severe liver dysfunction: (a) transaminase levels were more than 10 times the upper limit of normal; (b) total bilirubin levels were more than 2 times the upper limit of normal; (3) complicated with biliary system disease; (4) renal insufficiency (estimated glomerular filtration rate (eGFE) < 60 ml/min/1.73m^2^); (5) pregnancy and breastfeeding; and (6) age ≤ 18 years.

In addition, to clarify whether there were differences in the expression of HA in different populations, we also included 43 cases of non-SARS-CoV-2 pulmonary infection and 30 healthy individuals from physical examination as controls, according to the exclusion criteria above.

### Ethical considerations

This study was approved by the institutional review board of Beijing Ditan Hospital and conducted in accordance with the ethical standards laid down in the 1964 Declaration of Helsinki and its later amendments.

### Data collection

Demographic variables included age, sex, body mass index, personal history and clinical complications. COVID-19 related indicators included vaccination, number of infections, time from infection to recovery, viral shedding time, and prognosis. Laboratory tests were collected for liver function indicators (such as alanine aminotransferase and aspartate aminotransferase), renal function parameters (eGFR), routine blood tests (such as white blood cells and platelets), inflammation indicators (such as C-reactive protein and serum amyloid A), and coagulation indexes (such as D-dimer and fibrinogen). In addition, imaging features of pulmonary involvement attributed to infection were collected.

### Detection of serum HA

Serum samples were collected from the first day of admission, and HA levels were measured using a biochemical detector (HITACHI 7020, China).

### Study endpoints and definitions

The primary endpoint was the severity of SARS-CoV-2 infection. The secondary outcome measure was mortality.

The severity of SARS-CoV-2 infection was assessed by lung involvement based on chest CT. Mild COVID-19 infection was defined as no lung lesions and/or interstitial changes, whereas severe infection was defined as extensive ground-glass opacities, consolidation and/or ARDS.

### Statistical analysis

Continuous variables were expressed as mean ± standard deviation (M ± SD) or median (interquartile range) in case of skewed distribution. Differences between groups were analyzed by Student’s t test or Mann-Whitney U test. Categorical variables were presented as percentages (%) and their statistical analysis was performed by the Chi-square test or Fisher’s exact test. Logistic regression analysis was used to determine the factors influencing the severity of infection in the COVID-19 cohorts, and the results were presented as odds ratio (OR) and 95% confidence interval (CI). To understand the levels of HA expression in different subgroups, we controlled the confounding by performing a propensity score matching analysis. The propensity score was calculated using *a priori* logistic regression model based on covariates such as age and sex. Patients with COVID-19 were then matched in a 1:1 ratio to controls. Three logistic regression models were constructed. In model 1 (the crude model), no covariates were adjusted; in model 2, age and gender were adjusted; and in model 3, a total of 10 covariates were adjusted. Moreover, we divided the HA data into three groups according to the elevation of HA: < 90 ng/mL group, 90-130 ng/mL group, and > 130 ng/mL group. Discrimination performance of HA and other variables was assessed by receiver operating characteristic (ROC) curve analysis, and their areas under the curve (AUC) were compared using a nonparametric approach. Restricted cubic spline (RCS) was used to investigate the non-linear relationship between HA and mortality. We used smooth curve fitting and generalized additive models to identify the inflection point of the HA levels on the risk of death. Statistical analysis was performed with SPSS (version 26.0), and figures were generated using GraphPad Prism (version 9.4.1) and R (version 4.1.2). A two-sided P less than 0.05 was considered significant.

## Results

### Baseline characteristics

A total of 217 patients with COVID-19 were recruited and divided into two groups according to the cut-off value of HA: 80 cases in the HA < 90 ng/mL group and 137 cases in the HA ≥ 90 ng/mL group. Baseline demographic and clinical characteristics are shown in **Table 1**. Patients with high HA levels were older. The proportions of hypertension, diabetes mellitus and cerebrovascular disease were higher in the HA ≥ 90 ng/mL group (P < 0.05). In addition, significantly higher levels of fibrinogen, D-dimer, C-reactive protein, serum amyloid A and interleukin 6 were observed in patients with high HA levels (P < 0.05). However, patients in the high HA group had lower lymphocyte and hemoglobin levels (P < 0.05).

**Table 1:** Baseline characteristics of COVID-19 patients.

Characteristics	HA <90 ng/mL (n = 80)	HA ≥90 ng/mL (n = 137)	P value
Personal history			
Age (years)	56.7 ± 20.0	68.0 ± 15.1	<0.001
Female, n (%)	35 (43.8)	57 (42.3)	0.720
Body mass index (kg/m^2^)	23.7 ± 3.7	23.9 ± 4.1	0.729
Smoking, n (%)	28 (35.0)	56 (40.9)	0.391
Drinking, n (%)	27 (33.8)	57 (41.6)	0.251
Comorbidities, n (%)			
Hypertension	17 (21.3)	68 (49.6)	<0.001
Diabetes mellitus	11 (13.8)	41 (29.9)	0.007
Chronic kidney disease	3 (3.8)	14 (10.2)	0.087
Cerebrovascular disease	6 (7.5)	26 (19.0)	0.021
Cardiovascular disease	11 (13.8)	33 (24.1)	0.068
Liver disease	11 (13.8)	16 (11.7)	0.656
Liver function indicators			
Alanine aminotransferase, (U/L)	19.0 (13.5, 30.5)	21.0 (15.0, 33.0)	0.506
Aspartate aminotransferase, (U/L)	23.0 (17.0, 30.5)	26.0 (19.0, 40.5)	0.264
Total bilirubin, (μmol/L)	11.0 (7.3, 15.0)	10.0 (7.4, 13.0)	0.914
Renal function parameter			
eGFR, (ml/min/1.73m^2^)	93.3 ± 22.8	87.6 ± 29.7	0.142
Blood routine variables			
White blood cell, (10^9^/L)	6.7 ± 2.9	7.2 ± 3.2	0.215
Lymphocyte, (10^9^/L)	1.2 ± 0.8	0.9 ± 0.5	0.006
Hemoglobin, (g/L)	131.5 ± 27.2	122.7 ± 24.9	0.016
Platelet, (10^9^/L)	196.4 ± 94.9	178.8 ± 62.7	0.066
Coagulation indexes			
Prothrombin time, (s)	12.0 (11.0, 13.0)	12.0 (11.0, 13.0)	0.226
APTT, (s)	32.0 (29.0, 34.0)	31.0 (28.7, 34.0)	0.515
Fibrinogen, (mg/dL)	369.4 ± 110.7	436.1 ± 133.7	<0.001
D-dimer, (mg/L)	0.5 (0.4, 0.8)	0.8 (0.5, 1.4)	<0.001
Inflammatory indicators			
C-reactive protein, (mg/L)	12.0 (5.0, 34.5)	47.0 (15.0, 112.5)	<0.001
Serum amyloid A, (mg/L)	30.0 (8.0, 206.0)	241.0 (62.5, 395.5)	<0.001
Interleukin 6, (pg/mL)	10.0 (5.5, 20.8)	31.0 (11.5, 63.0)	<0.001

Values are number (percentage), median (interquartile range) or mean ± SD. COVID-19, coronavirus disease 2019; HA, hyaluronic acid; eGFR, estimated glomerular filtration rate; APTT, activated partial thromboplastintime.

### Imaging and clinical manifestations of SARS-CoV-2 infection between different HA groups

We noted that the high HA group had more cases of initial SARS-CoV-2 infection (83.9% vs. 70.0%, P = 0.015). There were significant differences on chest CT findings, including bilateral lung involvement, ground-glass opacities, consolidation and pleural effusion, between the two groups (P < 0.05). Although the incidence of pulmonary fibrosis was similar in both groups, the high HA group outnumbered the low HA group. High HA levels were associated with prolonged viral shedding time (11.0 (8.0, 15.0) vs. 8.0 (5.3, 14.0), P < 0.001) and recovery time (19.0 (9.5, 25.0) vs. 9.0 (5.2, 18.5), P < 0.001). Besides, the incidence of bacterial lung infection, respiratory failure, ARDS and death in the low HA group was better than in the high HA group (P < 0.05), as shown in **Table 2**.

**Table 2:** Imaging and clinical manifestations of SARS-CoV-2 infection between different HA groups.

Characteristics	Total (n = 217)	HA <90 ng/mL (n = 80)	HA ≥90 ng/mL (n = 137)	P value
COVID-19 vaccination, n (%)				
Unvaccinated/partially vaccinated	93 (42.8)	33 (41.3)	60 (43.8)	0.715
Fully vaccinated/booster doses	124 (57.2)	47 (58.8)	77 (56.2)	–
Numbers of infection, n (%)				
First infection	171 (78.8)	56 (70.0)	115 (83.9)	0.015
Re-infection	46 (21.2)	24 (30.0)	22 (16.1)	–
Chest CT findings, n (%)				
Bilateral lung involvement	188 (86.6)	58 (72.5)	130 (94.9)	<0.001
Ground-glass opacities	114 (52.5)	29 (36.6)	85 (62.0)	<0.001
Consolidation	67 (30.9)	9 (11.3)	58 (42.3)	<0.001
Pleural effusion	41 (18.9)	5 (6.3)	36 (26.3)	<0.001
Pulmonary fibrosis	9 (4.1)	1 (1.3)	8 (5.8)	0.159
Clinical course, (days), median (IQR)				
Viral shedding time	10.0 (7.0, 15.0)	8.0 (5.3, 14.0)	11.0 (8.0, 15.0)	<0.001
Recovery time	15.0 (9.0, 24.0)	9.0 (5.2, 18.5)	19.0 (9.5, 25.0)	<0.001
Outcomes, n (%)				
Combined bacterial infection	113 (52.0)	26 (32.5)	87 (63.5)	<0.001
Respiratory failure	55 (25.3)	12 (15.0)	43 (31.4)	0.007
Tracheal cannula	12 (5.5)	2 (2.5)	10 (7.3)	0.218
Acute respiratory distress syndrome	14 (6.5)	1 (1.3)	13 (9.5)	0.015
Death	19 (8.8)	1 (1.3)	18 (13.1)	0.003

Values are number (percentage) or median (interquartile range). SARS-CoV-2, severe acute respiratory syndrome coronavirus-2; HA, hyaluronic acid; COVID-19, coronavirus disease 2019; IQR, interquartile range. The symbol "-" indicates that the P value does not need to be reported.

### Risk factors of severe SARS-CoV-2 infection

There were 73 cases of severe SARS-CoV-2 infection. Univariate and multivariate logistic regression analysis were performed to investigate the risk factors for severe SARS-CoV-2 infection (**Table 3**). The results of single-factor logistic regression analysis showed that age, hypertension, diabetes mellitus, lymphocytes, fibrinogen, D-dimer, C-reactive protein, serum amyloid A and HA were all statistically significant (P < 0.05). When variables with P < 0.1 were included in the multivariate logistic regression analysis, we found that hypertension (OR = 2.023, 95%CI = 1.080-3.788), elevated D-dimer (OR = 2.156, 95%CI = 1.071-4.342), and high HA levels (OR = 4.540, 95% CI = 2.105-9.790) remained independent risk factors for severe infection (P < 0.05).

**Table 3:** Univariate and multivariate logistic regression analysis of severe infection.

Characteristics	Univariate analysis	P value	Multivariate analysis	P value
	OR (95% CI)		OR (95% CI)	
Age >65 years	2.745 (1.481-5.011)	0.001	1.539 (0.724-3.284)	0.269
Female	0.601 (0.337-1.074)	0.086	0.583 (0.301-1.130)	0.110
Body mass index >25 kg/m^2^	0.623 (0.334-1.161)	0.136		
Smoking	1.414 (0.784-2.551)	0.250		
Drinking	1.190 (0.649-2.181)	0.575		
Hypertension	2.912 (1.626-5.215)	<0.001	2.023 (1.080-3.788)	0.028
Diabetes mellitus	1.938 (1.057-3.553)	0.032	0.922 (0.444-1.913)	0.827
Chronic kidney disease	1.083 (0.384-3.055)	0.881		
Cerebrovascular disease	1.661 (0.774-3.565)	0.114		
Cardiovascular disease	1.107 (0.565-2.168)	0.768		
Fully vaccinated/booster doses	0.796 (0.452-1.404)	0.431		
White blood cell >10×10^9^/L	1.882 (0.917-3.865)	0.085	1.576 (0.670 -3.707)	0.298
Lymphocyte <1×10^9^/L	1.844 (1.040-3.268)	0.036	1.680 (0.862-3.272)	0.127
Hemoglobin <120g/L	1.184 (0.650-2.156)	0.581		
Platelet <100×10^9^/L	1.354 (0.527-3.475)	0.529		
Prothrombin time >12 s	1.318 (0.736-2.361)	0.353		
APTT >37 s	0.632 (0.220-1.814)	0.394		
Fibrinogen >400 mg/dL	2.324 (1.300-4.156)	0.004	1.605 (0.711-3.624)	0.255
D-dimer >0.5 mg/L	2.762 (1.413 -5.329)	0.002	2.156 (1.071-4.342)	0.031
C-reactive protein >20 mg/L	3.230 (1.730-6.032)	<0.001	1.167 (0.467-2.918)	0.741
Serum amyloid A >30 mg/L	2.688 (1.350-5.352)	0.005	1.154 (0.422-3.161)	0.780
Interleukin 6 >20 pg/L	1.148 (0.653-2.017)	0.632		
HA >90 ng/mL	5.959 (2.835-12.527)	<0.001	4.540 (2.105-9.790)	<0.001

OR, odds ratio; CI, confidence interval; APTT, activated partial thromboplastintime; HA, hyaluronic acid.

**Figure 1:**
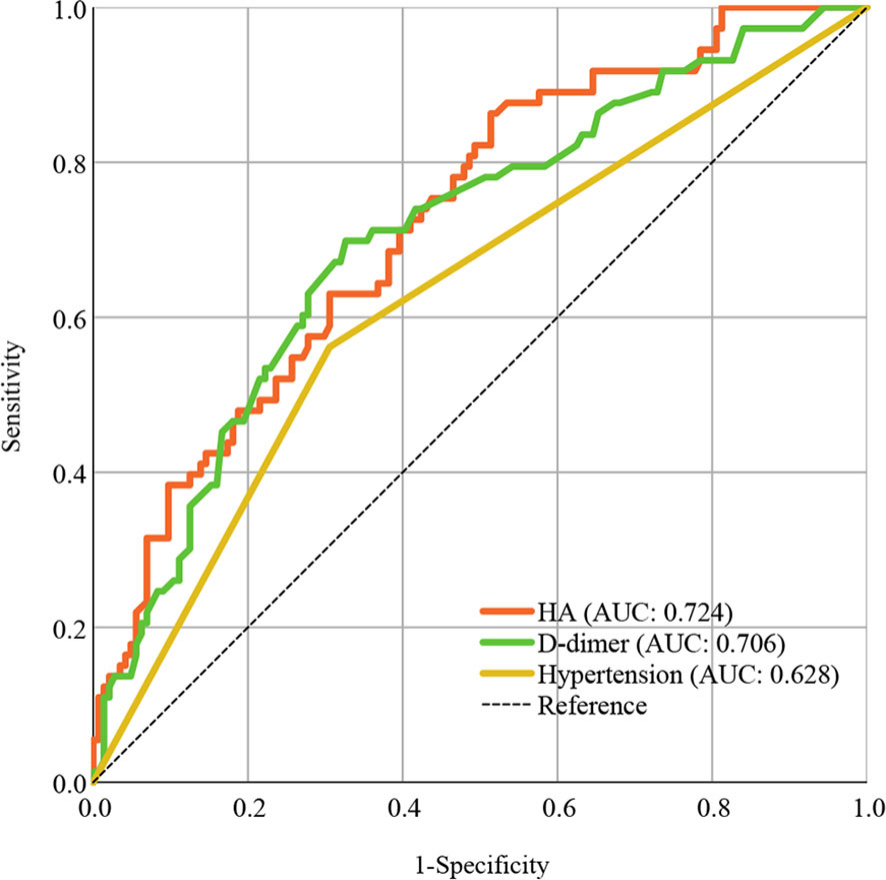
ROC curve analysis of HA compared to other risk factors in predicting COVID-19 progression. Areas under the ROC curve indicate model predictive power. ROC, receiver operating characteristic; AUC, areas under the curve; HA, hyaluronic acid; COVID-19, coronavirus disease 2019.

In the ROC curve analysis, HA (AUC: 0.724) presented better discrimination in predicting severe infection than D-dimer (AUC: 706) and hypertension (AUC: 0.628), as shown in **Figure 1**.

### HA levels and disease severity in subgroups

**Figure 2:**
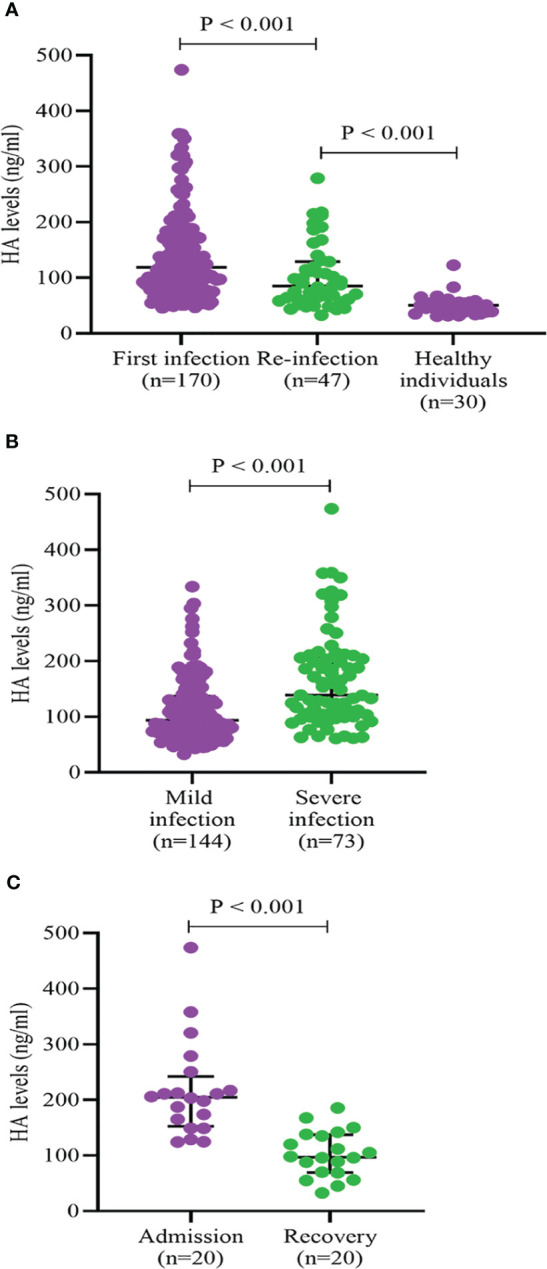
HA levels according to frequency of infection **(A)**, severity of infection **(B)**, and course of infection **(C)**. HA, hyaluronic acid. Differences between groups were analyzed by Mann-Whitney U test.

**Figure 3:**
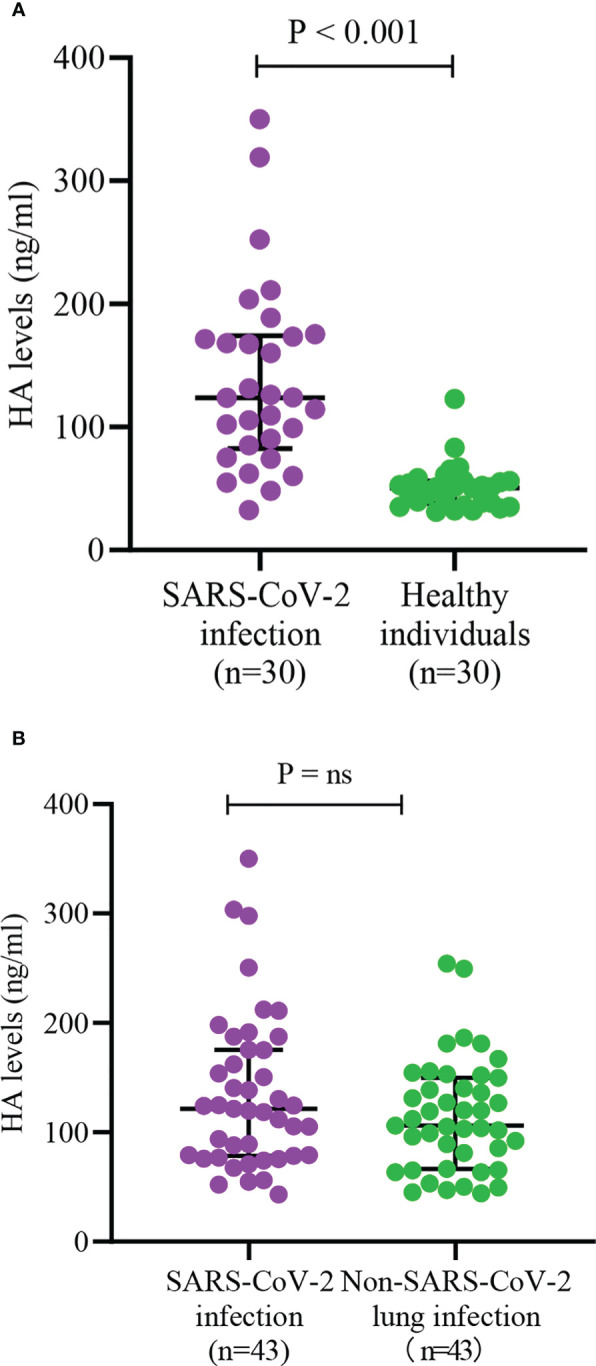
Serum HA levels between different status of infection **(A)** and different etiology of lung infection **(B)**. HA, hyaluronic acid; SARS-CoV-2, severe acute respiratory syndrome coronavirus-2. Differences between groups were analyzed by Mann-Whitney U test. "ns" means "not significant".

As shown in **Figure 2A**, we found that HA levels in COVID-19 re-infections were lower than in first infections (85.0 (61.1, 128.8) vs. 118.8 (79.5, 174.3), P < 0.001), but significantly higher than in healthy controls (85.0 (61.1, 128.8) vs. 50.5 (37.8, 66.8), P < 0.001). There was a higher proportion of severe infection (37.1% vs. 21.3%, P = 0.043) and death (10.6% vs. 2.1%, P = 0.069) in the first SARS-CoV-2 infections (**Supplementary Figures 1A, B**). The levels of HA were markedly higher in the severe COVID-19 group than that in the mild group (139.0 (101.9, 208.0) vs. 93.5 (68.6, 137.0), P < 0.001) (**Figure 2B**). To further assess the relationship between HA and the course of SARS-CoV-2 infection, we monitored the changes in HA levels before discharge in 20 severe COVID-19 cases. HA levels decreased with absorption of the pulmonary lesions (204.7 (152.9, 242.2) vs. 97.0 (69.3, 137.3), P < 0.001), but failed to fully return to normal levels (**Figure 2C**). We also analyzed HA levels in 30 age- and sex-matched healthy individuals and in 43 age-, sex- and infection severity-matched non-SARS-CoV-2 pulmonary infections. As expected, COVID-19 cases had higher HA concentrations than age- and gender-matched healthy individuals (123.9 (82.6, 174.1) vs. 50.5 (37.8, 66.8), P < 0.001) (**Figure 3A**). Although there was no significant difference on HA expression between COVID-19 and non-SARS-CoV-2 lung infections, the former had relatively higher HA levels (121.6 (78.5, 175.6) vs. 106.0 (66.5, 149.7), P = 0.244) (**Figure 3B**). This suggested that HA expression was also elevated in inflammatory respiratory disease.

### Association between HA and the risk of severe SARS-CoV-2 infection

As shown in **Table 4**, the results indicated that there was a positive association between HA and the risk of severe SARS-CoV-2 infection after adjusting for all covariates (OR = 1.009, 95%CI = 1.004-1.014, P < 0.001). In other words, elevated HA levels were associated with a higher risk of infection progression. In addition, we transformed the HA levels from a continuous to a categorical variable for analysis to investigate whether this correlation was stable. Patients with HA levels > 130 ng/mL had a 3.133-fold increased risk of severe infection compared to those with HA levels < 90 ng/mL (OR = 4.133, 95%CI = 1.740-9.818, P = 0.001; P for trend < 0.05).

**Table 4:** Relationship between HA and severe infection.

	Crude Model	P value	Model 1	P value	Model 2	P value
	OR (95%CI)		OR (95%CI)		OR (95%CI)	
HA	1.111 (1.107-1.116)	<0.001	1.010 (1.006-1.015)	0.002	1.009 (1.004-1.014)	<0.001
HA group						
<90 ng/mL	Reference		Reference		Reference	
90-130 ng/mL	4.529 (1.931-10.623)	0.001	4.060 (1.686-9.776)	0.002	3.094 (1.193-8.028)	0.020
>130 ng/mL	7.157 (3.247-15.856)	<0.001	6.004 (2.671-13.496)	<0.001	4.133 (1.740-9.818)	0.001
P for Trend		<0.001		<0.001		0.002

Crude model: non-covariates were adjusted. Model 1: age and gender were adjusted. Model 2: age, gender, hypertension, diabetes mellitus, white blood cells, lymphocytes, fibrinogen, D-dimer, C-reactive protein and serum amyloid A were adjusted. HA, hyaluronic acid; OR, odds ratio; CI, confidence interval.

**Figure 4:**
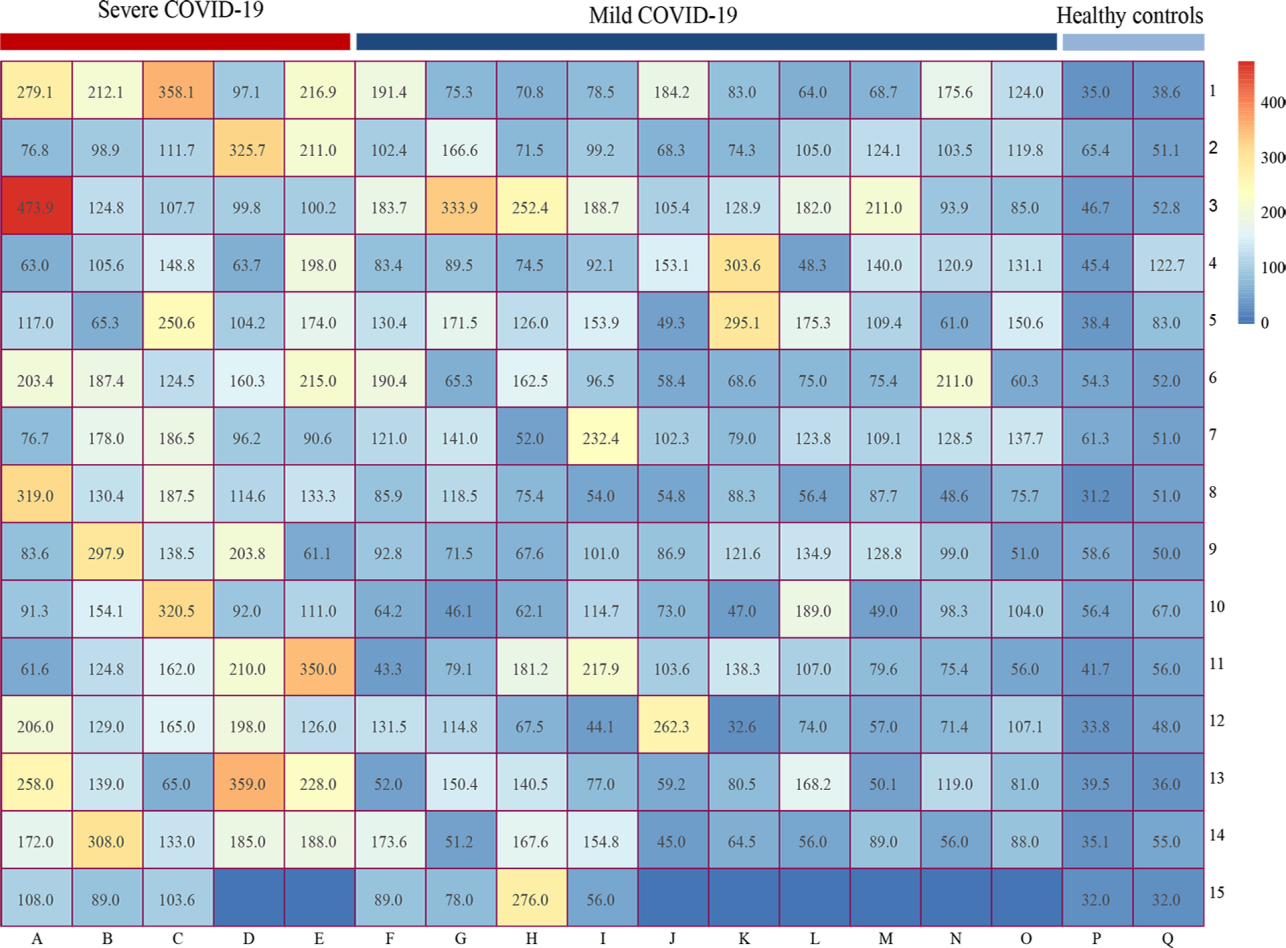
Matrix heatmap showing the expression of HA in COVID-19 patients and healthy controls. Heatmap mainly indicates the numerical size by different colors or shades. The abscissa represents different groups (A-E reflects the HA levels of severe COVID-19 group, F-O represents the HA levels of mild COVID-19 group, P-Q represents the HA levels of healthy control group), while the ordinate only represents the number of rows. HA, hyaluronic acid; COVID-19, coronavirus disease 2019.

The matrix heatmap showed the serum HA concentrations for COVID-19 individuals and healthy controls. Heatmap mainly indicates the numerical size by different colors or shades. The HA values in each group were presented randomly and no cluster analysis was performed. We observed that patients with severe SARS-CoV-2 infection had higher HA levels (**Figure 4**).

### The non-linear relationship between HA levels and risk of death

There were 19 deaths in our study population, 18 of which were in the high HA group (**Table 2**). Logistic regression analysis showed that lymphocytes, D-dimer, and HA (OR = 9.755, 95% CI = 1.216-78.245, P = 0.032) were independent predictors of death in COVID-19 cases (**Supplementary Table 1**). We also performed a four-point RCS to examine the association between HA and the risk of death in COVID-19 patients. There was a curvilinear relationship between HA and death risk, a forward ‘S’-shaped relationship. By calculating the inflection point, we observed that mortality increased with HA levels above 109 ng/mL. This is shown in **Supplementary Figure 2**.

### Influence of diabetes mellitus and atherosclerosis on HA levels

Previous reports have shown that diabetes mellitus and atherosclerosis may be associated with HA expression (Mine et al., 2006; Nieuwdorp et al., 2007). We analyzed HA levels in COVID-19 patients complicated by diabetes mellitus or coronary atherosclerotic disease and age-, sex- and infection severity-matched COVID-19 controls. However, HA levels were not significantly elevated in COVID-19 patients with diabetes mellitus (124.8 (92.0, 182.0) vs. 109.1 (75.3, 175.6), P = 0.202) or coronary atherosclerotic disease (122.4 (91.7, 174.6) vs. 114.4 (77.4, 171.1), P = 0.356) (**Supplementary Figures 3A, B**).

## Discussion

In this study, we found that (1) HA levels in SARS-CoV-2 infection cases were significantly higher than those in healthy individuals but similar to those in non-SARS-CoV-2 lung infections; (2) COVID-19 first-infected cases had higher HA levels and a higher proportion of severe infection than re-infections; (3) HA was positively associated with the severity of SARS-CoV-2 infection, including severe lung involvement, elevated inflammatory indicators, higher fibrinogen and D-dimer levels, prolonged viral shedding and recovery time, and death; (4) HA could be used as a predictor of severe COVID-19 (AUC: 0.724), with a 3.54-fold increased risk of severe infection in patients with high HA levels; and (5) HA expression was significantly downregulated with lung lesion absorption but did not fully return to normal levels.

Although the pathogenicity of SARS-CoV-2 strains has been reduced, their high transmission continues to threaten human health in the post-COVID-19 era (Singh et al., 2023). Nearly 80% of Chinese individuals were infected with COVID-19 in the omicron wave, with a significant proportion of them being re-infected (Liu and Wang, 2023). Lung involvement remains a major feature of SARS-CoV-2 infection in the post-COVID-19 era. Severe COVID-19 cases are characterized by pulmonary inflammation with progressive respiratory impairment (Mylvaganam et al., 2021; Hama Amin et al., 2022). It can severely complicate the clinical course and lead to an unfavorable prognosis for COVID-19 cases (Vianello et al., 2021). The assessment of lung lesions is mainly based on chest CT findings, but the test may be limited by poor compliance, radioactive nature or high cost, especially for outpatients. Thus, finding a simple and feasible serum biomarker for the early identification of individuals at high risk of SARS-CoV-2 infection progression is crucial and cost-effective. Previous reports have shown that HA is associated with SARS-CoV-2 infection. Early in the epidemic outbreak, Hellman et al. (2020) found HA exudates in the alveolar spaces of two autopsied COVID-19 lung tissues. Subsequent studies also demonstrated that human identical sequences of SARS-CoV-2 could promote HA upregulation during COVID-19 progression, and that inhibited HA expression may help reduce the severity of lung lesions (Li et al., 2022; Yang et al., 2022). It is important to emphasize that these studies were conducted during a period of intense viral pathogenicity and with small sample sizes. Therefore, the findings require further validation in large clinical trials in the post-COVID-19 era.

In this prospective study, we found that HA, hypertension and D-dimer were independent risk factors for severe COVID-19, but the predictive value of HA was superior to the other two markers. The AUC of HA discriminating 73 severe infection patients from 217 COVID-19 patients was as high as 0.724. In addition, some of other biomarkers have been reported to indicate mortality and prognosis in COVID-19, including lymphocytes, CRP and fibrinogen (Ponti et al., 2020). The changes in the serum concentrations of these biomarkers may be due to the subsequent cascade of inflammation and coagulation system rather than the virus itself (Yong et al., 2023). In contrast, human identical sequences of SARS-CoV-2 can directly promote the expression of HA by activating HA synthase (Li et al., 2022), thus HA may be able to predict infection progression early on admission and provide effective risk stratification. Moreover, we observed that viral shedding time and recovery time were prolonged in the high HA group. SARS-CoV-2 infection could upregulate HA expression (Li et al., 2022), thus HA levels may be positively correlated with viral load, with a correspondingly longer clinical course in patients with high HA levels. There was a higher incidence of respiratory failure, ARDS and death in the high HA group. Tissue and serum HA levels are elevated in response to inflammation and injury in the pulmonary infection process; the HA molecule is highly hygroscopic and can absorb up to thousands of times its molecular weight of water, which can contribute to pulmonary edema (Laurent and Fraser, 1996; Nagy et al., 2015a). In addition, HA has been proposed to be involved in the composition of liquid jelly in ARDS, the most severe form of acute lung injury and an vital contributor to death (Hällgren et al., 1989; Shi et al., 2020). These findings all suggest that individuals with elevated HA levels are at high risk of severe infection, prolonged clinical course and adverse outcomes.

Interestingly, we found higher levels of HA and a greater proportion of severe infection in COVID-19 first infections. HA is considered a member of the inflammatory factors, and its expression could be rapidly upregulated due to the strong inflammatory response in first infections (Avenoso et al., 2020). It is also possible that the virus-specific immune response, particularly antibodies and cellular immune memory, plays a critical role in controlling virus replication in re-infected individuals, resulting in a significant reduction in the activation of HA synthase (Yue et al., 2023). To further assess the correlation between HA and the course of SARS-CoV-2 infection, we monitored the changes in HA levels prior to discharge in 20 severe COVID-19 cases, and found that HA expression was significantly downregulated with infection recovery but did not fully return to normal levels. Recent research showed that SARS-CoV-2 can cause systemic infection and survive for months *in vivo* (Stein et al., 2022), thus the abnormal expression of HA may be associated with residual virus in organs and tissues during recovery. In addition, some COVID-19 patients were combined with bacterial pneumonia, the slower decline in HA levels may be due to a continued inflammatory process under the double whammy of virus and bacteria (Misra et al., 2015). Since HA has been used as an important diagnostic index to evaluate the degree of hepatic fibrosis, we speculate that persistently high HA levels may be associated with pulmonary consequences, such as lung consolidation and fibrosis (Bazdyrev et al., 2021; Kim and Seki, 2023). This evidence highlights the need to reassess HA levels within 3 to 6 months of infection, especially in patients with long COVID.

To date, no studies have compared HA expression between SARS-CoV-2 infection and common lung infections. This study showed that although there was no significant difference in HA expression between COVID-19 and age-, sex- and infection severity-matched non-SARS-CoV-2 pulmonary infections, the levels of HA were relatively higher in COVID-19 cases. Previous studies have shown that HA participates in a variety of physiological processes, including inflammation and autoimmunity, and therefore can be elevated in non-SARS-CoV-2 lung infections (Nagy et al., 2015b; Sauer et al., 2022). In addition, the HA levels of our COVID-19 patients were measured on the first day of admission, when the virus had just invaded the body and a low viral load might not significantly upregulate HA expression. Admittedly, we only included 43 non-SARS-CoV-2 lung infections, and the small sample size may lead to insufficient statistical power. HA expression may increase as the virus continues to replicate *in vivo*, so future studies need to monitor dynamic changes of HA throughout the course of COVID-19 and compare them with non-SARS-CoV-2 infections.

Importantly, we also observed a non-linear, forward ‘S’-shaped relationship between HA levels and death, with mortality increasing significantly when HA levels were higher than 109 ng/mL. In other words, elevated HA levels were independently associated with death in COVID-19 patients. This suggests that clinicians need to be aware of the risk of death predicted by elevated HA in critical COVID-19 patients. Previous studies have shown that HA metabolism is associated with diabetes mellitus and atherosclerosis, but all have focused on patients without pulmonary infection (Mine et al., 2006; Nieuwdorp et al., 2007). As there were more cases of diabetes mellitus and coronary atherosclerotic disease in the high HA group, we then investigated the possible relationship between HA expression and diabetes mellitus or coronary atherosclerotic disease in COVID-19 patients. However, both diabetes mellitus and atherosclerosis had no effect on the expression of HA in COVID-19 patients. The findings, which were inconsistent with previous reports, may be related to the small number of patients and the different study contexts. It is also possible that HA expression may be affected by several uncontrollable confounders in clinical practice, such as chronic liver complications and individual heterogeneity (Heldin et al., 2019; Moran-Salvador et al., 2019). More likely, SARS-CoV-2 promotes the accumulation of HA by activating HA synthase, thereby masking the minor HA fluctuations caused by diabetes mellitus or atherosclerosis (Li et al., 2022).

Some limitations should be noted. First, the study was conducted in a single-center, which means that the findings need to be further validated in large scale multi-center studies. Second, there was a case selection bias due to different research backgrounds. Our study population is predominantly elderly. In the post-COVID-19 era, the majority of people with SARS-CoV-2 infection are able to recover spontaneously due to reduced viral pathogenicity, and those who seek medical help tend to be older and in poor health. In addition, we did not evaluate the effect of HA expression blockers on COVID-19 infection, which could add a valuable dimension to the results. This may be related to the following: 1) current COVID-19 treatment regimens are relatively mature and well established, and drugs with uncertain efficacy may have some side effects; 2) most hospitalized patients are elderly and have multiple chronic comorbidities, and anti-infective treatment may be complementary to symptomatic supportive care. Finally, we focused mainly on HA levels at the time of presentation, and did not analyze changes in HA during the course of COVID-19 and within 3 to 6 months after recovery.

## Conclusion

HA was associated with SARS-CoV-2 infection, and it could be used as a simple alternative biomarker to predict SARS-CoV-2 infection progression and assist early clinical decisions in the post-COVID-19 era. In addition, HA levels and the proportion of severe infections increased significantly in first COVID-19 infections. This suggests that clinicians need to be concerned about the risk of infection progression predicted by elevated HA levels in COVID-19 patients, particularly in the first infections.

## Data availability statement

The original contributions presented in the study are included in the article/**Supplementary Material**. Further inquiries can be directed to the corresponding author.

## Ethics statement

The studies involving humans were approved by Institutional Review Board of Beijing Ditan Hospital. The studies were conducted in accordance with the local legislation and institutional requirements. As all COVID-19 patients have blood tests to assess the severity of the infection on admission, we collect serum samples left over from these tests for further research. Written informed consent for participation was not required from the participants or the participants’ legal guardians/next of kin in accordance with the national legislation and institutional requirements.

## Author contributions

YLi: Writing – original draft, Data curation, Formal analysis, Investigation, Methodology, Writing – review & editing. XC: Data curation, Formal analysis, Writing – original draft. NZ: Data curation, Investigation, Writing – original draft. YLin: Data curation, Investigation, Writing – original draft. XL: Project administration, Resources, Supervision, Validation, Writing – review & editing.
