# Supplementary figures and images for: Elevated hyaluronic acid levels in severe SARS-CoV-2 infection in the post-COVID-19 era
Source: Front Cell Infect Microbiol. 2024 Feb 8;14:1338508. doi: 10.3389/fcimb.2024.1338508 (PMC10881864; doi:10.3389/fcimb.2024.1338508)

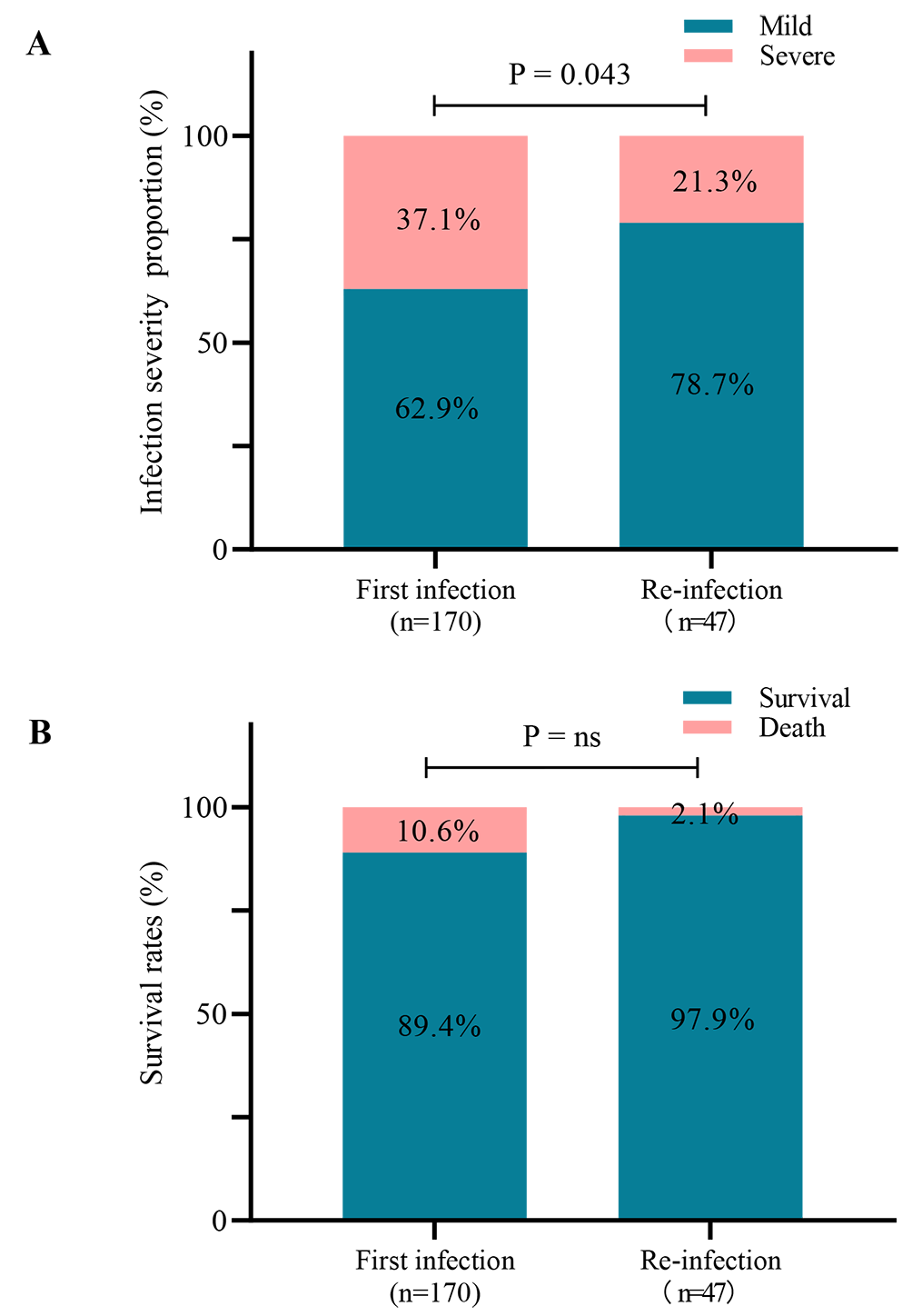

Supplement: Supplementary file 1 [file Image_1.tif]

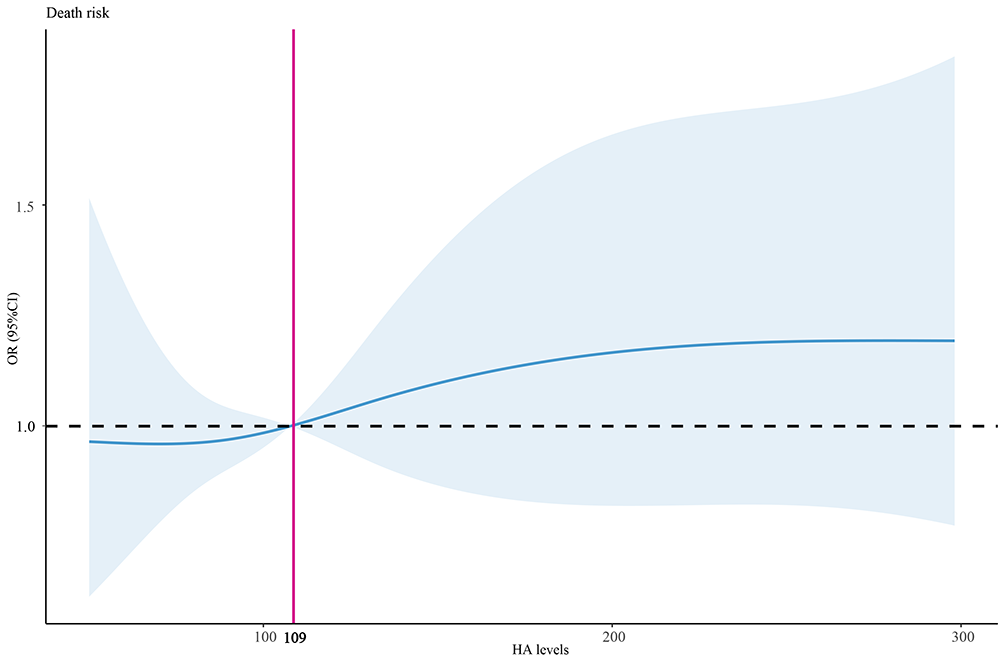

Supplement: Supplementary file 2 [file Image_2.tif]

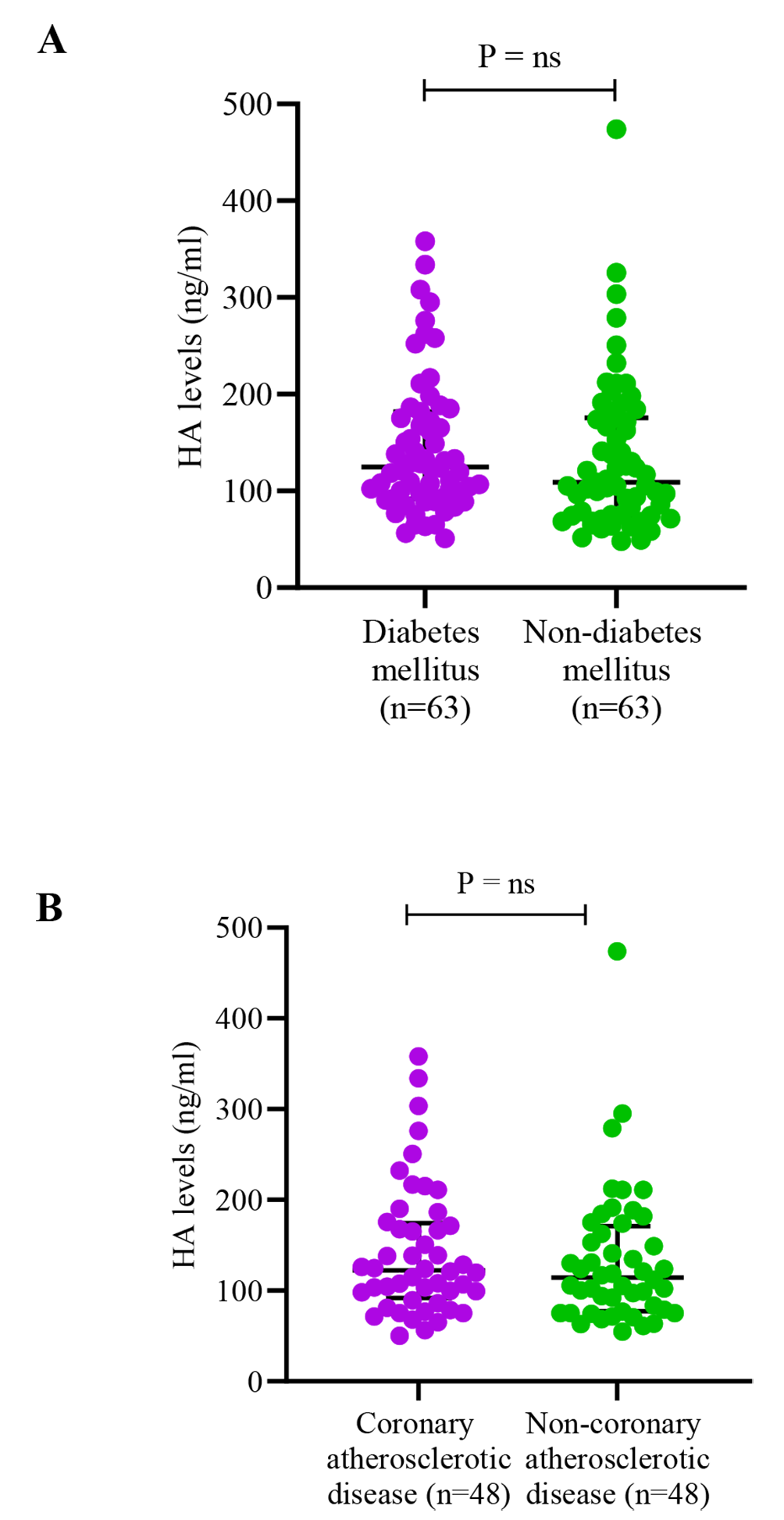

Supplement: Supplementary file 3 [file Image_3.tif]
