# Supplementary material for: Elevated hyaluronic acid levels in severe SARS-CoV-2 infection in the post-COVID-19 era
Source: Front Cell Infect Microbiol. 2024 Feb 8;14:1338508. doi: 10.3389/fcimb.2024.1338508 (PMC10881864; doi:10.3389/fcimb.2024.1338508)
Supplement: Supplementary file 4 [file Table_1.docx]

**Supplementary Table 1 Univariate and multivariate logistic regression analysis of death risk**

| Characteristics | Univariate analysis |  |  | Multivariate analysis |  |
| --- | --- | --- | --- | --- | --- |
|  | OR (95% CI) | P value |  | OR (95% CI) | P value |
| Age >65 years | 2.240 (0.777-6.458) | 0.136 |  |  |  |
| Female | 0.429 (0.149-1.235) | 0.117 |  |  |  |
| Body mass index >25 kg/m^2^ | 0.944 (0.343-2.598) | 0.912 |  |  |  |
| Smoking | 3.943 (1.480-10.508) | 0.006 |  | 3.927 (1.355-11.383) | 0.055 |
| Drinking | 2.817 (1.087-7.301) | 0.033 |  | 0.337 (0.037-3.044) | 0.332 |
| Hypertension | 2.935 (1.106-7.789) | 0.031 |  | 2.080 (0.667-6.483) | 0.207 |
| Diabetes mellitus | 1.479 (0.544-3.950) | 0.435 |  |  |  |
| Chronic kidney disease | 0.632 (0.079-5.046) | 0.665 |  |  |  |
| Cerebrovascular disease | 2.262 (0.754-6.787) | 0.145 |  |  |  |
| Cardiovascular disease | 0.389 (0.087-1.745) | 0.218 |  |  |  |
| Fully vaccinated/booster doses | 1.034 (0.399-2.683) | 0.945 |  |  |  |
| White blood cell >10*10^9/L | 1.853 (0.624-5.505) | 0.267 |  |  |  |
| Lymphocyte <1*10^9/L | 6.022 (1.701-21.32) | 0.005 |  | 4.499 (1.199-16.885) | 0.026 |
| Hemoglobin <120 g/L | 2.070 (0.800-5.353) | 0.133 |  |  |  |
| Platelet <100*10^9/L | 1.176 (0.251-5.505) | 0.836 |  |  |  |
| Prothrombin time >12 s | 2.172 (0.842-5.603) | 0.108 |  |  |  |
| APTT >37 s | 1.176 (0.251-5.505) | 0.836 |  |  |  |
| Fibrinogen >400 mg/dL | 2.256 (0.824-6.173) | 0.113 |  |  |  |
| D-dimer >0.5 mg/L | 10.742 (1.405-82.130) | 0.022 |  | 11.110 (1.374-89.823) | 0.024 |
| C-reactive protein >20 mg/L | 7.083 (1.594-31.481) | 0.010 |  | 1.781 (0.284-11.146) | 0.538 |
| Serum amyloid A >30 mg/L | 8.797 (1.149-67.344) | 0.036 |  | 1.777 (0.316-9.991) | 0.514 |
| Interleukin 6 >20 pg/L | 1.821 (0.689-4.819) | 0.227 |  |  |  |
| HA >90 ng/mL | 11.950 (1.564-91.319) | 0.017 |  | 9.755 (1.216-78.245) | 0.032 |

Abbreviations: OR, odds ratio; CI, confidence interval; APTT, activated partial thromboplastintime; HA, hyaluronic acid.
