# Supplementary material for: Elevated hyaluronic acid levels in severe SARS-CoV-2 infection in the post-COVID-19 era
Source: Front Cell Infect Microbiol. 2024 Feb 8;14:1338508. doi: 10.3389/fcimb.2024.1338508 (PMC10881864; doi:10.3389/fcimb.2024.1338508)
Supplement: Supplementary file 5 [file Table_2.docx]

**Supplementary Figure Legends**

**Supplementary Figure 1**

Incidence of severe COVID-19 (A) and death (B) according to infection frequency. COVID-19, coronavirus disease 2019.

**Supplementary Figure 2**

Association between HA and death in COVID-19 patients using a RCS regression model. HA, hyaluronic acid; COVID-19, coronavirus disease 2019; RCS, restricted cubic spline; OR, odds ratio; CI, confidence interval.

**Supplementary Figure 3**

Influence of diabetes mellitus (A) and coronary atherosclerotic disease (B) on HA levels in COVID-19 cases. HA, hyaluronic acid; COVID-19, coronavirus disease 2019.
